# Analysis of body composition with bioelectrical impedance analysis in different subtypes of pulmonary fibrosis

**DOI:** 10.1038/s41598-026-39350-5

**Published:** 2026-03-06

**Authors:** Katharina Buschulte, Ben Ehrhart, Bernhard Kötter, Katharina Abbasi Dezfouli, Oliver Weinheimer, Katharina Cremer, Christoph Lederer, Philipp Höger, Markus Polke, Felix F. J. Herth, Judith Brock

**Affiliations:** 1 Center for Interstitial and Rare Lung Diseases, Department of Pulmonology and Critical Care Medicine, Thoraxklinik University of Heidelberg, , Heidelberg, Germany; 2 Translational Research Center Heidelberg (TLRC), Member of the German Centre for Lung Research (DZL), Heidelberg, Germany; 3 Diagnostic and Interventional Radiology, Heidelberg University Hospital, Heidelberg, Germany; 4 Department of Pulmonology and Critical Care Medicine, Thoraxklinik, University of Heidelberg, Heidelberg, Germany

**Keywords:** Acute exacerbation, BIA, Body composition, ILD, Phase angle, Pulmonary fibrosis., Diseases, Immunology, Medical research

## Abstract

Patients with Idiopathic Pulmonary Fibrosis (IPF) often have an unfavourable body composition, characterized by a reduced phase angle (PhA) as an index of cellular health and associated with increased mortality. Little is known about body composition in other subtypes of pulmonary fibrosis (PF). In this single-centre, prospective study, bioelectrical impedance analysis (BIA) was used to assess body composition in patients with PF. In addition, metabolic parameters, lung function, exercise capacity and quality of life (QoL) questionnaires were collected. The fibrosis index (FIBI) was calculated by quantitative computed tomography. A total of 90 patients (57.8% male, mean age 70.8 ± 8.9 years) was analysed. Lung function was mildly impaired with a mean forced vital capacity (FVC) of 76.8 ± 21.4% predicted and mean diffusion capacity for carbon monoxide single breath (DLCO-SB) of 49.5 ± 16.0% predicted. The mean FIBI was 22.5% (SD 10.2). Main diagnoses were systemic autoimmune rheumatic diseases-associated ILD (*n* = 26, 29.9%), unclassified ILD (*n* = 19, 21.1%) and fibrosing hypersensitivity pneumonitis (*n* = 14, 15.6%). 22 patients (24.4%) received steroids and 26 (28.9%) other immunosuppressants. 22 patients (24.4%) experienced ≥ 1 acute exacerbation (AE). The mean body weight was 84.5 ± 18.2 kg with a mean body mass index (BMI) of 28.6 ± 5.7 kg/m^2^. One patient was underweight, and 31 patients (34.4%) had lost weight during the previous 6 months. BIA showed unfavourable values compared to healthy controls: body fat ↑ (28.9 ± 8.0%), extracellular mass/ body cell mass (ECM/BCM) index ↑ (1.2; IQR 1.0, 1.4), PhA ↓ (4.9 ± 1.0°), cell percentage ↓ (45.4 ± 6.3%) and fat-free mass index ↑ (19.9 ± 2.7 kg/m^2^). Correlation analyses showed a moderate correlation between PhA and FVC (*p* < 0.001). No relevant correlations were found between DLCO-SB, walking distance, FIBI, QoL and BIA parameters. Patients with ≥ 1 AE had a significantly worse PhA (*p* = 0.030). In addition, a sex difference was observed with significantly worse values of PhA for women compared to men (*p* = 0.036). Patients with PF had an unfavourable body composition with reduced PhA, reduced cell percentage and elevated ECM/BCM index. Significantly lower PhA values were found in patients with ≥ 1 AE and in women. Following longitudinal and interventional confirmation of our results, future research aimed at improving body composition and patient outcomes for those with PF could be conducted.

## Introduction

Interstitial lung diseases (ILD) are a group of over 200 mainly chronic conditions that affect the lung parenchyma due to inflammation and/or fibrosis^1^. Like one of the most common subtype, Idiopathic Pulmonary Fibrosis (IPF), other types of pulmonary fibrosis (PF) can also be chronic and progressive^2^. An international definition of progressive pulmonary fibrosis (PPF) has been published that considers radiological evidence of progression as well as worsening of respiratory symptoms and forced vital capacity (FVC) decline^3^. Progression is associated with an increased risk of mortality^4^. Typical symptoms include exertional dyspnoea, dry cough and fatigue^5^, leading to a reduction in quality of life (QoL)^6^. Therefore in addition to medical treatment, a holistic approach should be adopted to positively influence the disease course and alleviate symptoms^7^.

In addition to symptom burden and QoL, ILD severity also affects body composition. ILD patients with more severely impaired lung function showed a significantly lower muscle mass and higher fat mass^8^. Weight and body mass index (BMI) alone are insufficient to describe body composition. Bioelectrical impedance analysis (BIA) is a standardized and established tool to analyse body composition by assessing parameters for muscle mass, fat mass and body water based on a three-compartment model. It also provides information on cell health and training state^9^. To date, most studies using BIA have been performed in IPF. It has been shown that a quarter of IPF patients with a normal to obese BMI have an unfavourable body composition^10^. Furthermore, fat-free mass index (FFMI) was an independent predictor of survival in patients with IPF^11^. In PF, FFMI and severe malnutrition were significant predictors of overall survival regardless of disease severity^12^. To date, less data is available describing body composition in patients with other subtypes of PF.

The primary aim of this exploratory study was to investigate the body composition analysed by BIA in different subtypes of PF as well as gaining insight in physical activity, nutritional status and dietary behavior in PF. The secondary objectives were to identify factors influencing body composition in PF and to establish correlations with the severity of fibrosis.

## Methods

### Study design

This single-centre prospective observational study was carried out from April 2024 to May 2025 at the Thoraxklinik Heidelberg. Inclusion criteria were the diagnosis of PF confirmed by a multidisciplinary team (MDT), age over 18 years and the ability to give informed consent. Exclusion criteria included electronic implanted medical devices (e.g. pacemaker, implantable cardioverter defibrillator and brain stimulator), a lack of mobility, active cancer, neurodegenerative disorder/ muscle diseases (e.g. muscular dystrophy, amyotrophic lateral sclerosis, motor neuron disease), and the presence of tuberculosis or other non-pulmonary wasting disease with progressive weight loss. The study was approved by the Ethics Committee of the Medical Faculty of the University of Heidelberg, Germany (S-754/2023) and performed in accordance with the Declaration of Helsinki.

### Data collection

A total of 90 patients with incident or prevalent PF without antifibrotic therapy were included in this analysis. The following data was collected prospectively: demographic data (age, sex), the exact ILD diagnosis and time since diagnosis, ILD therapies and time since therapy, comorbidities (diabetes mellitus [DM], osteoporosis, and thyroid disease), and medication (e.g. diuretics, cholesterol, osteoporosis, diabetes or thyroid medications). In addition, we asked for acute exacerbations (AE) within the last 12 months, weight course within the last 6 months, frequency of physical activity per week, muscle exercise training (yes/no), respiratory therapy (yes/no), physiotherapy (yes/no), and dietary behaviour (no specialities/vegan/vegetarian/protein supplementation). Furthermore, metabolic parameters such as HbA1c, cholesterol, HDL- and LDL-cholesterol, triglycerides, and thyroid parameters were determined. Results from blood gas analysis, lung function (FVC, forced expiratory volume in one second [FEV_1_], total lung capacity [TLC] in l and %) and diffusing capacity for carbon monoxide single breath (DLCO-SB), exercise capacity (walking distance from 6-minutes walking distance [6-MWD]) and QoL questionnaires (modified Medical Research Council Dyspnea Scale [mMRC], St. Georges Respiratory Questionnaire [SGRQ]) were recorded. High-resolution computed tomography (HRCT) scans were analysed for fibrosis pattern as well as for the extent of fibrosis. The fibrosis index (FIBI) was obtained using YACTA (yet another CT analyzer)^13^, a quantitative computed tomography (CT) software (version: Yacta v3.0.0.21). FIBI was calculated as percentage of the segmented lung voxels ≥ − 700 HU^14^, voxels labeled as vessel are excluded. The height and weight of all patients were measured by a standardized, calibrated body weight scale and length gauge of “seca” (seca Deutschland, medical measurement systems and scales, Hamburg, Germany). BMI was calculated in kg/m^2^.

### Bioelectrical impedance analysis

**Fig. 1:**
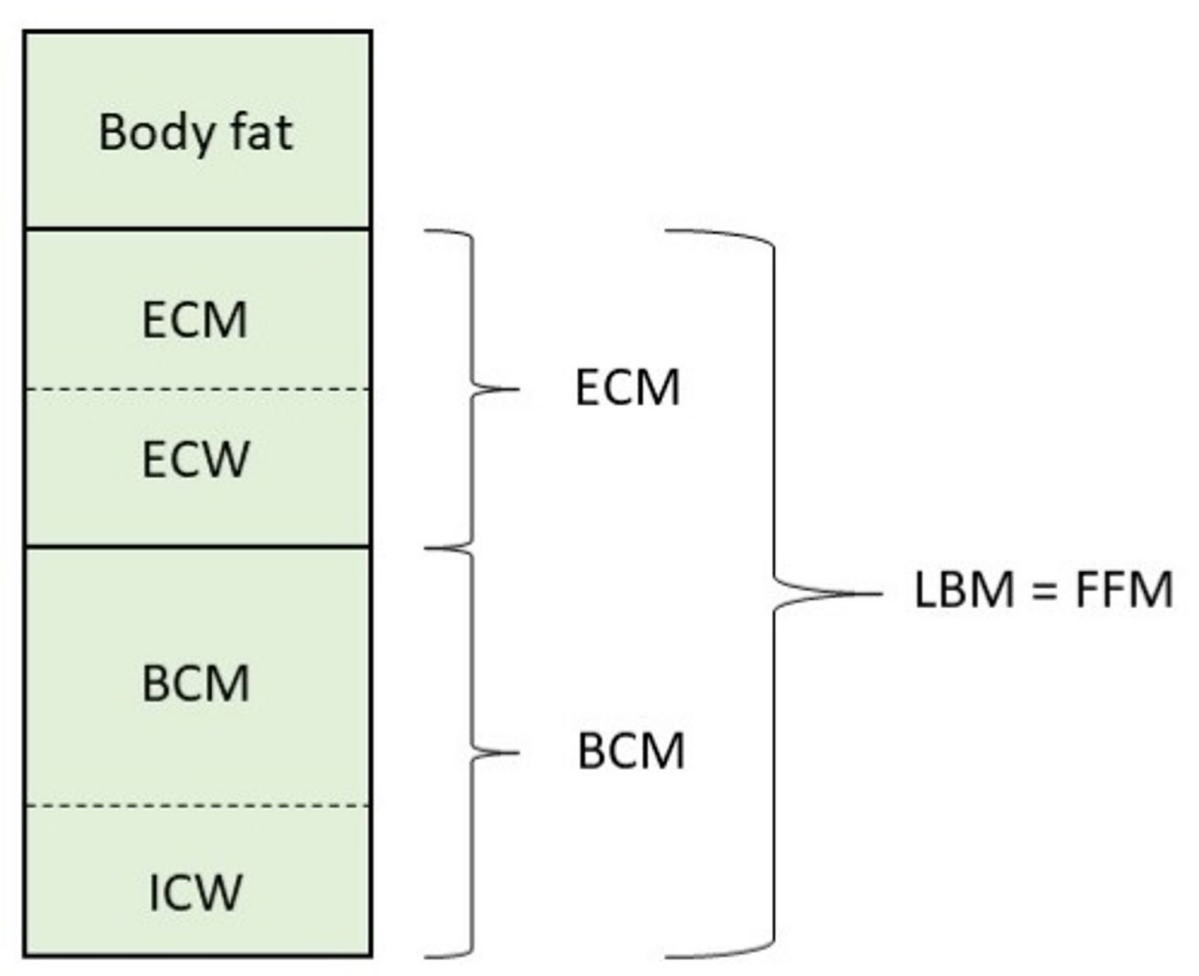
Three-compartment model with ECW and ICW of bioelectrical impedance analysis, adapted from^9^. Phase-sensitive multifrequency measurement with additional resistance determination at low frequencies (5 kHz) allows total body water (TBW) to be divided into intracellular water (ICW) and extracellular water (ECW). BCM, body cell mass; ECM, extracellular mass; ECW, extracellular water; FFM, fat-free mass; ICW, intracellular water; LBM, lean body mass.

We used the “Nutriguard-MS” multifrequency BIA device (Version 2.0, 2019, Data Input GmbH, Pöcking, Germany) to assess body composition based on a three-compartment model (Fig. 1^9^ . Two electrodes were attached to the hand and foot of the dominant side of the patient’s body. Measurements were performed at different frequencies (5 kHz, 50 kHz and 100 kHz) according to the instructions for use (supine position with the limbs not touching each other, after fasting >6 h, no sports and alcohol in the last 12 h). Transferring the data to the Nutriguard-MS software (version 2.0, 2019, Data Input GmbH) allows the following BIA parameters to be calculated: basal metabolic rate in kilocalories (kcal), body fat (BF) in kg and %, cell percentage in %, body cell mass (BCM; sum of all metabolic active cells, consisting of muscle and organ cell mass) in kg and %, extracellular mass (ECM; consisting of interstitium, bone, connective tissue) in kg and %, ECM/BCM-index, extracellular (ECW) and intracellular water (ICW) in litres, fat-free mass (FFM = BCM+ECM) in kg, fat-free mass index (FFMI) in kg/m^2^, lean body mass (LBM), phase angle (PhA) in °, total body water (TBW) in litres.

The phase angle (PhA) was of particular interest as it is a general parameter of cell density and membrane integrity, providing information about the condition and health of the cells. PhA is directly proportional to BCM. The TBW, which consists of 43% extracellular and 57% intracellular water, is primarily determined by BCM. The cell percentage is the proportion of BCM cells within the LBM, and is therefore a measure of nutritional and training status^9,15^. A healthy body composition is characterised by high phase angle, high cell percentage, and low ECM/BCM index. Various pathologies can cause an unfavourable body composition, manifesting as a deviation from the aforementioned BIA parameters. This can lead to a disruption of cell integrity and cell health, characterised by a reduced PhA. Additionally, shifts between compartments can occur, such as an expansion of the ECM due to extracellular water retention (ECW ↑)^9,15^. A combination of a reduced PhA, a reduced cell percentage and an elevated ECM/BCM index is indicative of particularly unfavourable body composition.

### Primary and secondary outcomes

The primary outcome parameter was PhA and other BIA parameters in a group of patients with various subtypes of PF. Secondary parameters of interest were correlations of body composition with basic characteristics such as sex, lung function parameters and exercise capacity, FIBI, QoL and AE.

### Data analysis

The statistical analysis of the collected data was mainly descriptive in order to describe the basic characteristics of the cohort. Data are presented as absolute numbers and percentages, medians/ interquartile range (IQR) were used for non-normally distributed variables, and means/ standard deviation (SD) for normally distributed variables. BIA parameters were examined for the whole group as well as separately by sex. For the main target criteria “PhA” and “BMI,” Pearson correlations with basic characteristics were performed. Correlations were considered high for *r* = 0.7–0.9, moderate for *r* = 0.4–0.69, weak for *r* = 0.3–0.39, and not relevant for *r* < 0.3^16,17^. All analyses are of explorative nature and have no confirmative value. For this reason, we did not perform a sample size or power calculation. T-test for independent samples were used for an analysis of differences of BIA parameters and BMI in different subgroups (male/female sex, AE yes/no, steroids yes/no, ILD subtype). A Bonferroni correction was applied to analyse the group differences in ILD subtypes due to the increased risk of type I error. Nevertheless, all results were interpreted cautiously due to the increased risk of type I and II errors. In addition, we performed a multivariate linear regression analysis, adjusting for various factors, including age, sex, disease severity (as measured by FVC and FIBI), ILD treatment (steroid and immunosuppressive therapies) and comorbidities such as DM, osteoporosis and thyroid disease, with PhA and BMI as parameters of greatest interest for body composition serving as the dependent variables. In order to examine the BIA parameters in relation to sex, sex-specific standard values were used as a basis. The percentage deviation from these standard values was calculated in each case and then compared with each other (T-test for independent samples). Statistical significance was defined as a two-sided at p value < 0.05 for all analyses. Excel and SPSS version 29.0.0.0 were used for all analyses.

## Results

### Study population

A total of 90 patients were included in this study. The baseline characteristics are shown in Table 1. Most patients (57.8%) were male with a mean age of 70.8 years (SD 8.9). The mean FVC was 76.8% predicted (SD 21.4) and the mean DLCO-SB was 49.5% predicted (SD 16.0). Most patients were diagnosed with systemic autoimmune rheumatic diseases-associated ILD (SARD-ILD; *n* = 26, 29.9%), followed by unclassified ILD (uILD; *n* = 19, 21.1%) and fibrosing hypersensitivity pneumonitis (fHP; *n* = 14, 15.6%). The mean time since diagnosis was 11.8 months. 22 patients (24.4%) received steroids and 26 (28.9%) other immunosuppressants. 22 patients (24.4%) experienced one or more AE: 12 patients experienced one, 9 patients experienced two, and one patient experienced more than two. The ILD pattern in HRCT was distributed as follows: 62.2% fibrosing NSIP, 17.8% typical and probable UIP, 17.8% indeterminate UIP, and 2.2% other. The mean FIBI derived from CT scans was 22.5% (SD 10.2). Certain comorbidities were recorded: 19 patients had osteoporosis (21.2%), 17 DM type 2 (18.9%), 15 hypothyroidism (16.7%), and 2 hyperthyroidism (2.2%).

**Table 1:** Baseline characteristics of all included patients.

Characteristics		Total (n=90)
Male Sex, n (%)		52 (57.8)
Age [years], mean (SD)		70.8 (8.9)
FVC [% predicted] (n=89), mean (SD)		76.8 (21.4)
FVC [l] (n=89), mean (SD)		2.8 (0.9)
TLC [% predicted] (n=89), mean (SD)		72.7 (18.8)
TLC [l] (n=89), mean (SD)		4.5 (1.3)
DLCO-SB [% predicted] (n=87), mean (SD)		49.5 (16.0)
DLCO/VA [% predicted] (n=86), mean (SD)		72.7 (19.7)
Walking distance in 6-minute walk [m] (n=78), mean (SD)		347.8 (97.5)
QoL, mean (SD)	mMRC	1.6 (1.3)
	SGRQ-Symptoms	43.6 (25.4)
	SGRQ-Activity	49.7 (26.7)
	SGRQ-Impacts	26.4 (19.7)
	SGRQ-Total	36.6 (21.3)
Smoking status	Active, n (%)	4 (4.4)
	Quit, n (%)	55 (61.1)
	Never, n (%)	31 (34.4)
	Pack years, mean (SD)	16.4 (19.7)
ILD-diagnosis, n (%)	IPF	7 (7.8)
	fHP	14 (15.6)
	SARD	26 (28.9)
	uILD	19 (21.1)
	iNSIP	9 (10.0)
	Other*	15 (16.7)
Time since ILD-diagnosis [months], mean (min; max)		11.8 (0; 127)
Therapy with steroids, n (%)		22 (24.4)
Time since therapy with steroids [months] (n=22), median [IQR]		11.5 (14.2)
Therapy with other immunosuppress-ants, n (%)		26 (28.9)
Time since therapy with immunosuppressants [months] (n=26), median [IQR]		36.5 (69.8)
Long term oxygen therapy, n (%)		15 (16.7)
Acute exacerbations, n (%)	0	68 (75.6)
	1	12 (13.3)
	2	9 (10.0)
	>2	1 (1.1)
Fibrosis index [%], mean (SD)		22.5 (10.2)

*The group of other ILD diagnoses comprises subgroups with a low case count: 3 interstitial pneumonia with autoimmune features IPAF, 3 drug-induced ILD, 3 Respiratory bronchiolitis-ILD RB-ILD, 2 asbest fibrosis, 1 interstitial lung abnormality [ILA], 1 inflammatory bowel disease-associated ILD, 1 idiopathic haemorrhagic syndrome, 1 occupation-related ILD.

DLCO-SB, diffusion capacity for carbon monoxide single breath; DLCO/VA, diffusing capacity divided by the alveolar volume; fHP, fibrosing hypersensitivity pneumonitis; FVC, forced vital capacity; ILD, interstitial lung disease; iNSIP, idiopathic non-specific interstitial pneumonia; IPF, idiopathic pulmonary fibrosis; SARD, systemic autoimmune rheumatic diseases; SD, standard deviation; TLC, total lung capacity; uILD, unclassified ILD.

### Activity level, dietary behaviour and body weight

The patients experienced reduced exercise capacity with a mean walking distance of 347.8 m (SD 97.5, Table 1). 31 patients (34.4%) engaged in physical activity more than 3 times a week, 27 did so 2–3 times a week (30.0%), and 19 did so once a week (21.1%). 13 patients (14.4%) reported that they did not engage in any physical activity. Only 5.6% of patients underwent physiotherapy, 3.3% respiratory therapy, and 1.1% muscle exercise training. Almost all patients (98.9%) showed no special dietary behavior; one patient was vegetarian. The mean body weight was 81.5 kg (SD 18.2) and the mean BMI 28.6 kg/m^2^ (SD 5.7). One patient (1.1%) was underweight with a BMI < 18.5 kg/m^2^, while 31 patients (34.4%) had lost weight during the previous 6 months. 76,7% of patients were overweight (*n* = 69) with a BMI > 25 kg/m^2^, and 9 (10%) had gained weight in the last 6 months.

#### Body composition

BIA measurement showed unfavorable values compared to age- and sex-matched, normal weight healthy controls with reduced parameters for cell percentage and basal metabolic rate, and elevated parameters for body fat, ECM/BMC index, total body weight, lean body mass, ECM, and FFMI *(*Table 2*).* BCM showed low values for women and mildly elevated levels for men. The mean PhA was reduced to 4.9 ° (SD 1.0); while 80% of patients (*n* = 72) showed deviations below the norm. The mean PhA was 5.2 ° in men (SD 1.0) and 4.5 ° in women (SD 0.8). A group of healthy individuals aged 60–69 was used for comparison because there are no comparative values available for ILD patients aged 70 and over.

**Table 2:** Functional parameter and BIA measurement results of all patients in comparison to average values of healthy persons.

	All (*n* = 90)	Men (*n* = 52)	Average values for healthy men^a^	Women (*n* = 38)	Average values for healthy women^a^
Baseline characteristics					
Age	70.8 (8.9)	70.9 (9.8)	–	70.7 (7.5)	–
Weight loss, n (%)	31 (34.4)	15 (28.8)	–	16 (42.1)	–
Functional parameter					
FVC [% predicted] (*n* = 89), mean (SD)	76.8 (21.4)	77.1 (20.0)	–	76.4 (23.4)	–
FVC [l] (*n* = 89), mean (SD)	2.8 (0.9)	3.2 (0,9)	–	2.2 (0.7)	–
DLCo-SB [% predicted] (*n* = 87), mean (SD)	49.5 (16.0)	47.8 (16.3)	–	52.0 (15.5)	–
Walking distance in 6-minute walk [m] (*n* = 78), mean (SD)	347.8 (97.5)	348.9 (108.5)	–	346.37 (83.6)	–
Fibrosis index [%], mean (SD)	22.5 (10.2)	20.9 (8.1)	–	24.7 (12.3)	–
BIA measurement results					
Measurement at right side of the body, n (%)	86 (95.6)	50 (96.2)	–	36 (94.7)	–
Body weight [kg], mean (SD)	84.5 (18.2)	86.0 (16.6)	72.7 (6.8)	75.4 (18.7)	63.2 (5.9)
BMI [kg/m2], mean (SD)	28.6 (5.7)	28.4 (4.5)	–	28.9 (7.1)	–
Body fat [kg], mean (SD)	24.1 (10.5)	21.9 (8.2)	14.1 (4.5)	27.1 (12.6)	18.6 (4.0)
Body fat [%], mean (SD)	28.9 (8.0)	24.9 (5.2)	19.2 (5.3)	34.5 (7.9)	29.2 (4.8)
TBW [l], mean (SD)	41.9 (8.6)	46.8 (7.2)	42.9 (4.0)	35.3 (5.5)	32.7 (2.9)
LBM [kg], mean (SD)	57.3 (11.8)	63.9 (9.8)	58.6 (5.4)	48.3 (7.5)	44.6 (3.9)
ECM [kg], mean (SD)	31.0 (6.0)	33.7 (5.4)	29.0 (4.0)	27.4 (4.7)	22.7 (2.6)
BCM [kg], mean (SD)	26.3 (7.5)	30.2 (6.7)	29.6 (4.0)	20.9 (4.6)	22.0 (2.8)
ECM/BCM index, median (IQR)	1.2 (0.4)	1.1 (0.3)	1.00 (0.2)	1.3 (0.3)	1.05 (0.17)
Cell percentage [%], mean (SD)	45.4 (6.3)	47.0 (6.2)	50.5 (4.9)	43.2 (5.9)	49.2 (4.1)
Basal metabolic rate [kcal], mean (SD)	1445.9 (236.0)	1469.6 (211.7)	1550 (125)	1276.8 (145.9)	1310 (90)
Phase angle [°], mean (SD)	4.9 (1.0)	5.2 (1.0)	5.8 (1.1)	4.5 (0.8)	5.5 (0.8)
ECW [l] (*n* = 89), mean (SD)	18.5 (5.4)	20.6 (5.0)	–	15.4 (4.4)	–
ICW [l], mean (SD)	23.7 (4.8)	26.2 (3.9)	–	20.3 (3.8)	–
FFM [kg], mean (SD)	57.3 (11.8)	63.9 (9.8)	–	48.3 (7.5)	–
FFMI [kg/m2], mean (SD)	19.9 (2.7)	21.1 (2.6)	16.7–19.8^b^	18.3 (2.1)	14.6–16.8^b^

^a^ Average values for healthy and normal weight (BMI 19–24.9 kg/m2) persons, age matched for 60–69 years (Source: Data Input, based on analysis of *n* = 29.409 women and *n* = 2224 men)^9^.

^b^ There are no absolute normal values for FFMI, but these values are considered normal.

BCM, body cell mass; BIA, bioelectrical impedance analysis; BMI, body mass index; DLCO-SB, diffusing capacity for CO-single breath; ECM, extracellular mass; ECW, extracellular water; FFM, fat-free mass; FFMI, fat-free mass index; FVC, forced vital capacity; ICW, intracellular water; LBM, lean body mass; SD, standard deviation; TBW, total body water.

### Factors influencing body composition

**Fig. 2:**
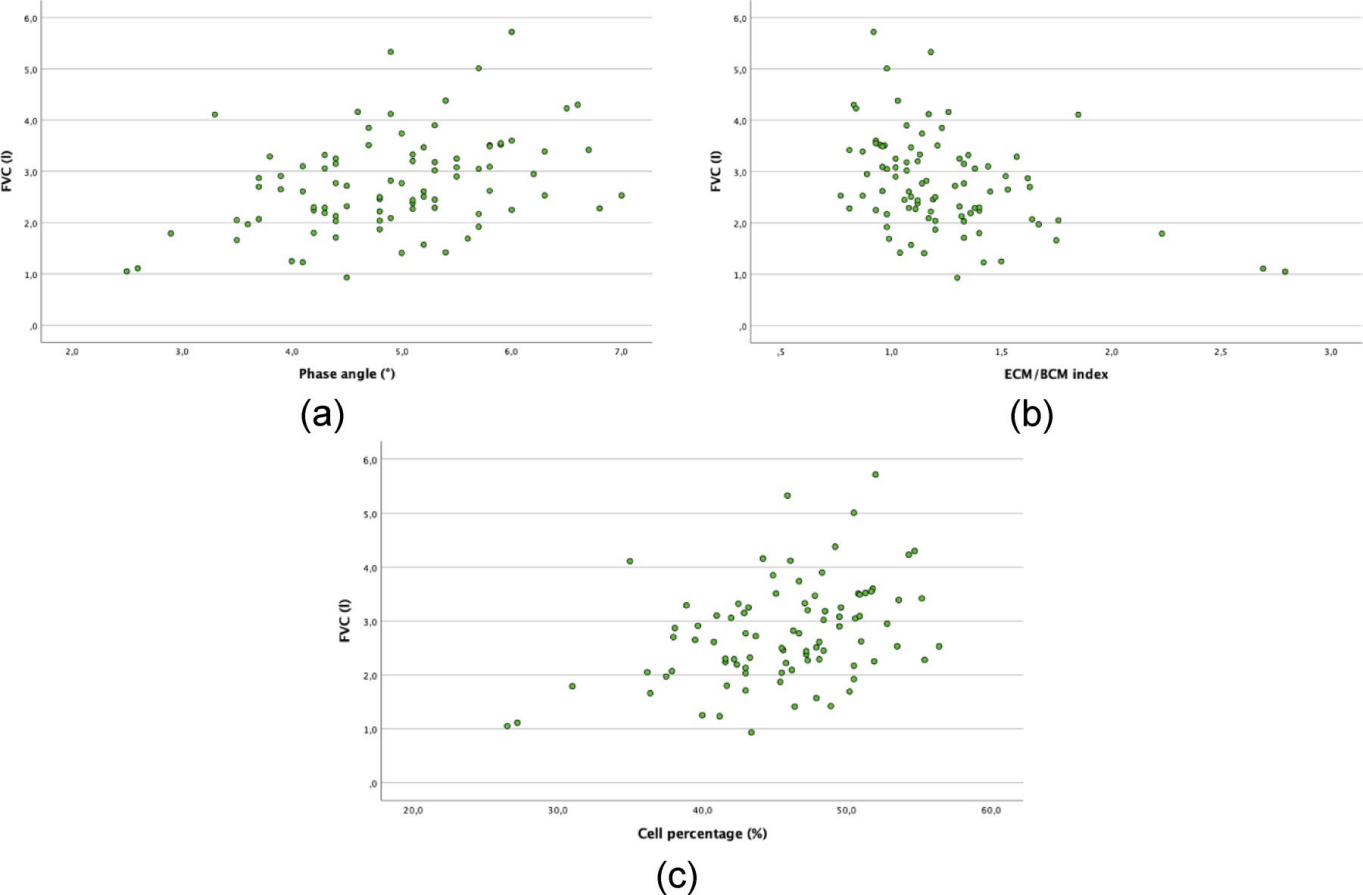
Correlation analyses for FVC in litres and phase angle (**a**), ECM/BCM index (**b**), and cell percentage (**c**). BCM, body cell mass; ECM, extracellular mass; FVC, forced vital capacity.

Secondary analyses focused on factors influencing body composition. A higher PhA was observed at higher FVC values (*r* = 0.388, *p* < 0.001; Fig. 2a). Higher values for ECM/BCM index (*r* = 0.403, *p* < 0.001; Fig. 2b) and cell percentage (*r* = 0.401, *p* < 0.001; Fig. 2c) were also observed with higher FVC. Patients who lost weight had a notably lower PhA (*r* = 0.398, *p* < 0.001). No relevant correlations (*r* < 0.3) were found between diffusing capacity (DLCO-SB and diffusing capacity divided by the alveolar volume [DLCO/VA]), walking distance, long term oxygen therapy, QoL, and BIA parameters (Table 3).

**Table 3:** Correlation of phase angle and BMI with baseline characteristics (Pearson correlation).

	Phase angle		BMI	
	Pearson correlation	*p*	Pearson correlation	*p*
FVC [l]	0.388**	< 0.001	− 0.082	0.443
FVC [% predicted]	0.119	0.269	− 0.151	0.157
DLCO-SB [% predicted]	0.076	0.484	0.055	0.611
DLCO/VA [% predicted]	− 0.017	0.876	0.179	0.099
Long term oxygen therapy	− 0.159	0.135	0.001	0.990
Fibrosis index [%]	− 0.037	0.730	0.169	0.111
mMRC	− 0.210*	0.047	0.241*	0.022
SGRQ-Symptoms	− 0.056	0.603	0.211*	0.046
SGRQ-Activity	− 0.172	0.105	0.226*	0.032
SGRQ-Impacts	− 0.197	0.063	0.113	0.288
SGRQ-total	− 0.173	0.103	0.174	0.100
ILD diagnosis	0.080	0.453	0.018	0.867
Time since ILD-diagnosis [months]	− 0.066	0.539	0.011	0.921
BMI [kg/m2]	0.163	0.124	–	–
Phase angle [°]	–	–	0.163	0.124
Weight trend	0.398**	< 0.001	− 0.109	0.308
Walking distance in 6-minute walk [m]	0.236*	0.037	− 0.158	0.167
Physical activity per week	0.148	0.163	− 0.036	0.740
Acute exacerbations	− 0.255*	0.015	0.035	0.742
Long-term oxygen therapy	− 0.159	0.135	0.001	0.990
Therapy with steroids	− 0.158	0.136	− 0.059	0.580
Therapy with other immunosuppressants	− 0.036	0.737	− 0.077	0.472

* The correlation is significant at the 0.05 level (2-sided).

**The correlation is significant at the 0.01 level (2-sided).

BMI, body mass index; DLCO-SB, diffusing capacity for CO-single breath; DLCO/VA, diffusing capacity divided by the alveolar volume; FVC, forced vital capacity; ILD, interstitial lung disease; mMRC, Modified Medical Research Council Dyspnea Scale; SGRQ, St. Georges Respiratory Questionnaire.

**Fig. 3:**
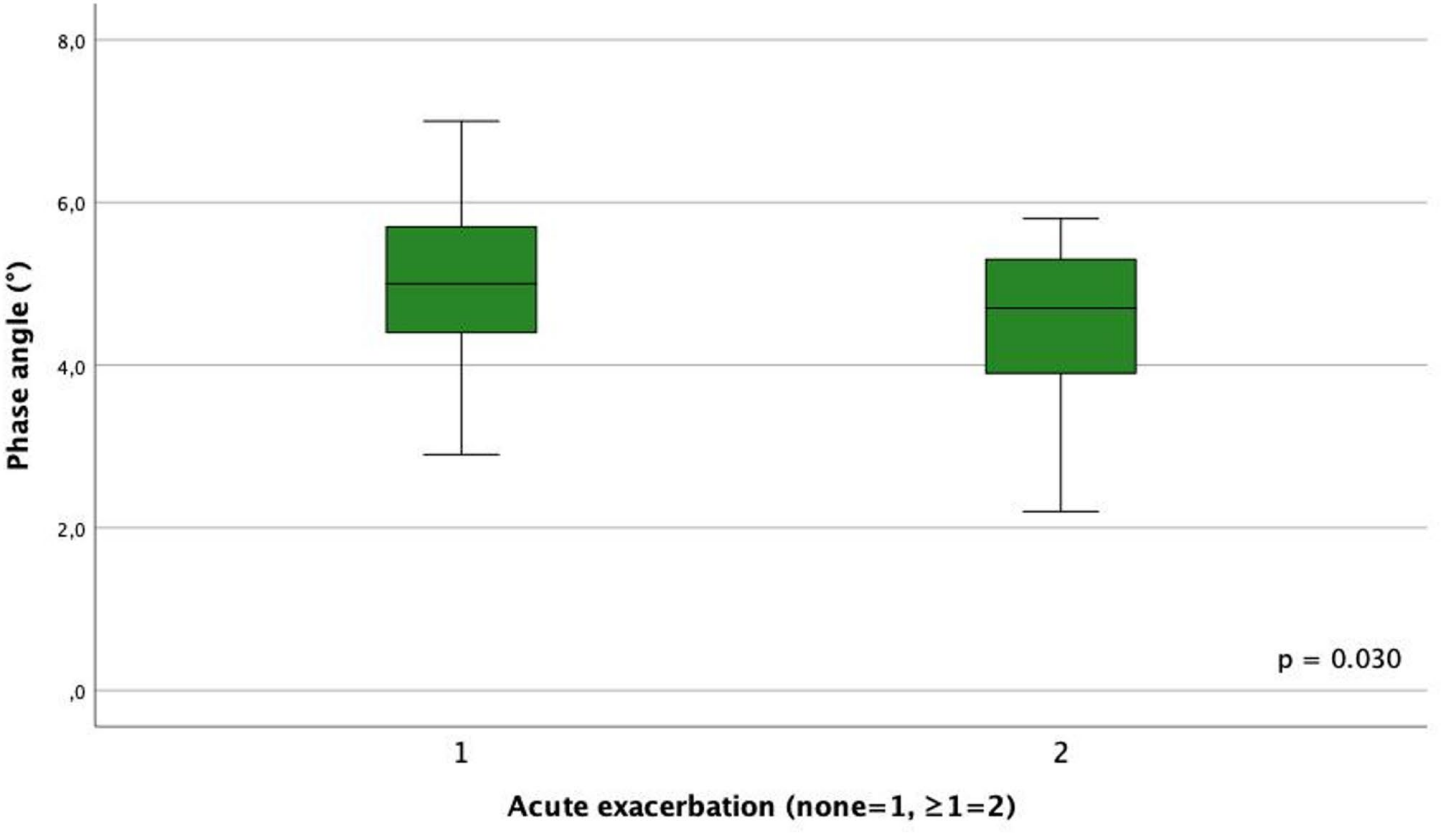
Phase angle in patients with and without acute exacerbations.

The following subgroup analyses are of explorative nature and based on small sample sizes. With regard to the different subtypes of ILD, patients with uILD had the lowest PhA at 4.4 ° (SD 1.1) and patients with iNSIP had the highest PhA at 5.4 ° (SD 0.5). There were no significant differences between the ILD subtypes. Patients with one or more AE had with 4.5 ° (SD 1.1) a significantly worse PhA (*p* = 0.030; Fig. 3). The same correlation was found between ≥ 1 AE and cell percentage (*p* = 0.043), but not between ≥ 1 AE and ECM/BCM index. In addition, women showed a significantly worse PhA compared to men. The percentage deviation of PhA from the average values for healthy women was 17.9% and for men 10.0% (*p* = 0.036). BMI showed no relevant correlations to ILD subtype, AE or sex.

After adjusting for potential confounders such as age, sex, disease severity (FVC and FIBI), ILD treatment (steroids and immunosuppressants) and comorbidities (DM, osteoporosis and thyroid disease), poorer PhA was observed in patients with one or more AE (*p* = 0.049, Table 4). BMI showed no significant correlations with sex, AE, steroid therapy or ILD subtype.

**Table 4:** Results of univariate analyses and multivariate linear regression analysis with phase angle and BMI.

		Phase angle				BMI			
		Mean	SD	p (univariate)	p_adj_ (multivariate)	Mean	SD	p (univariate)	p_adj_ (multivariate)
Sex	male	5.1	1.0	0.001	0.020	28.4	4.5	0.709	0.657
	female	4.5	0.8			28.9	7.1		
Acute exacerbations	yes	4.5	1.1	0.030	0.049	28.5	5.7	0.751	0.932
	no	5.0	0.9			28.9	6.1		
Steroid therapy	yes	4.6	1.0	0.165	0.287	28.8	5.8	0.580	0.228
	no	4.9	0.9			28.0	5.7		
ILD subtype	IPF	5.0	0.7	0.066^+^	0.696	27.8	6.3	0.708^+^	0.924
	fHP	5.1	0.9			29.8	5.7		
	SARD	4.7	1.1			27.3	7.0		
	uILD	4.4	1.1			29.6	5.8		
	iNSIP	5.4	0.5			29.4	3.6		
	Other*	5.2	0.7			28.2	3.9		

Univariate analyses were performed using t-test for independent samples. A multivariate model was used to adjust for age, sex, disease severity (FVC and FIBI), ILD treatment (steroid and immunosuppressive therapies) and comorbidities (DM, osteoporosis and thyroid disease), with PhA and BMI serving as the dependent variables.

*The group of other ILD diagnoses comprises subgroups with a low case count: 3 interstitial pneumonia with autoimmune features IPAF, 3 drug-induced ILD, 3 Respiratory bronchiolitis-ILD RB-ILD, 2 asbest fibrosis, 1 interstitial lung abnormality [ILA], 1 inflammatory bowel disease-associated ILD, 1 idiopathic haemorrhagic syndrome, 1 occupation-related ILD.

^+^with Bonferroni correction.

BMI, body mass index; fHP, fibrosing hypersensitivity pneumonitis; FVC, forced vital capacity; ILD, interstitial lung disease; iNSIP, idiopathic non-specific interstitial pneumonia; IPF, idiopathic pulmonary fibrosis; SARD, systemic autoimmune rheumatic diseases; SD, standard deviation; uILD, unclassified ILD.

The mean FIBI was 22.5% (SD 10.2), ranging from a minimum of 9.3% to a maximum of 61.9%. FIBI and FVC showed a moderate correlation (*r* = 0.620, *p* < 0.001), while no correlation between FIBI and PhA or other BIA parameters were shown. There was also no relevant association between FIBI and BMI. Subanalyses by sex showed a weak correlation between FIBI and PhA for men (*r* = 0.315, *p* = 0.023), but this was not observed in the group of women.

## Discussion

Our results revealed an unfavourable body composition in ninety patients with different subtypes of PF, characterised by reduced PhA and cell percentage, as well as an increased ECM/BCM index. We also identified possible influencing factors, including AE and female sex. There was no correlation between fibrosis index and body composition. To the best of our knowledge, no studies have currently investigated these factors influencing body composition in PF.

We compared our BIA measurement results with the average values for healthy, normal-weight individuals aged 60–69 years^9^. It should be noted that our cohort was slightly older, with an average age of 70.8 years. While the age difference is relatively small, it could still have affected the extent to which the BIA parameters deviated from those of the comparison group. Published cohorts of elderly individuals show that there are age-related decreases in PhA and reactance, particularly in men, with notable changes occurring after the age of 70^18^. However, the heterogeneity of BIA measurement methods (e.g. DXA, magnetic resonance imaging, and other multicomponent approaches) and device types must be considered as this often makes it impossible to compare different cohorts. Other variables include electrode type, adherence to standardised measurement protocols, and the stored software algorithms. Reference values should only be used if they have been validated using the same reference method^19^. In our study, we therefore used the comparative data for healthy subjects provided by the manufacturer of the BIA device, unfortunately this did not include any subjects over the age of 70^9^. Unfortunately, reference values for patients with ILD and/or in this age are not established.

Our cohort comprises various subtypes of PF, including SARD-ILD, uILD, fHP, iNSIP and IPF. Lung function parameters revealed a mild respectively moderate impairment of FVC and DLCO. To date, the use of BIA measurement to assess body composition in patients with ILD has been most extensively studied in patients with IPF. Although PhA as an index parameter for cell density and cellular health is the most well-established impedance parameter^20^, it is not always reported. Machado et al. demonstrated abnormally low PhA in one-fourth of included patients with IPF. Patients with low PhA had worse lung function, exercise capacity and QoL^10^. Reduced PhA was also observed in other fibrosing ILDs in both women and men by Rinaldi et al.^12^. Our values for PhA were even lower than those of the Rinaldi et al. cohort, although the functional values of both groups were similar^12^. However, this may be due to the different ILD subtypes included in the studies. Additionally, BIA measurement can reveal information about an unfavourable shift between compartments. Rinaldi et al. also demonstrated that a reduced FFMI negatively affects exercise capacity, regardless of disease severity^12^. Another study also found a reduced FFMI in IPF patients, and FFMI was also shown to be an independent predictor of survival^11^. Our results showed an elevated FFMI, unlike the studies mentioned. This was primarily due to an increase in ECM resulting from extracellular water retention and therefore a quantitative increase in lean mass.

To better interpret BIA results, the corresponding compendium of the software suggests classifying certain values. Both the PhA and the ECM/BCM index in our cohort are rated as “poor” compared to standard values^9^. This highlights the extent of the unhealthy body composition of our cohort of patients with PF, which is a key issue that needs to be addressed. Interestingly, low PhA is often found even in people with a normal or even an overweight BMI^10^. Our data also showed no correlation between BMI and PhA or other BIA parameters. Therefore, it can be concluded that BMI alone is insufficient for assessing nutritional status.

In ILD, FVC is considered an important prognostic factor^5^ and is therefore used, along with clinical and radiological parameters, to diagnose PPF^3^. We demonstrated a correlation between PhA and FVC as well as a correlation of FVC with cell percentage and ECM/BCM index. Low FVC is therefore associated with worse body composition. A correlation between PhA and pulmonary function parameters (FEV_1_ and FVC) was also observed in other diseases, such as chronic obstructive pulmonary disease (COPD)^21^. Other studies in PF have also demonstrated a correlation between disease severity and body composition. Guler et al. examined body composition using dual-energy X-ray absorptiometry. They found correlations between ILD severity (FVC and DLCO) and muscle mass as well as percentage body fat^8^, especially in men.

In contrast, our study could not reveal associations between DLCO and body composition, neither between walking distance and body composition. The fibrosis index averaged at 22.5%, but ranging from a minimum of 9.3% to a maximum of 61.9%. Similarly, our study was unable to establish a correlation with body composition. When interpreting these results, the risk of type II errors must be taken into account. However, the possibility of a temporal dissociation between structural fibrosis and systemic and/or metabolic changes should be considered. Multi-omic profiling in systemic sclerosis-associated ILD revealed that metabolic changes in lipid metabolism are associated with ILD progression. These changes may either precede the extent of structural fibrosis or develop independently of it^22^. Similar findings have been demonstrated in IPF and other fibrosing ILD^23^. Furthermore, it can be assumed that body composition is not solely determined by the degree of fibrosis and functional impairment, but that other systemic or metabolic factors also play a role. A recent study showed that haematological parameters were linked to lean mass. Additionally, canonical analyses identified a “metabolic–cellular integrity” axis linking zinc and iron status to PhA and fluid balance. The authors emphasise that sarcopenia and fluid shifts play a key role in older patients. Therefore, as previously mentioned, BMI is insufficient for assessing nutritional status, and PhA is as a much more promising parameter of cellular health^24^. QoL was reduced in our cohort, particularly with regard to symptoms and activity limitations, but no correlation with PhA or other BIA parameters was found. Studies on various types of cancer showed a good correlation between BIA and health status as well as QoL^25^. However, this correlation has not yet been investigated in ILD.

Another secondary finding of our study was a sex difference showing that women had poorer PhA compared to men. Overall, sex naturally influences body composition and PhA. This is due to natural differences between the sexes, such as women having higher fat percentage and men having higher muscle mass^26^. Therefore, we used the deviation from sex-specific standard values as a basis here, and still found a significantly greater deviation in women, indicating a poorer body composition.

In terms of ILD subtypes, the greatest reduction in PhA was observed in patients with uILD. No comparable data from other studies is available. However, various studies have already demonstrated that the prognosis for uILD is worse than for other ILD^27^. Patients with uILD who have detectable fibrosis on HRCT, as in the patients included in our study, have a poor prognosis similar to patients with IPF^28^.

Nearly 25% of patients had at least one AE within the last 12 months. AE have a poor outcome in PF^29^, and must therefore be regarded as a serious event. Here, we were able to show that patients with at least one AE had poorer PhA and cell percentage, but not ECM/BCM index. It should be noted, however, that these secondary subgroup analyses, like the aforementioned analyses of ILD subtypes, are based on relatively small patient numbers and should therefore not be overinterpreted. Nevertheless, our findings are in line with a study of COPD, in which an increased number of AE in COPD patients was found to correlate with higher extracellular water ratios and lower PhA compared with stable COPD patients^30^. These results illustrate that patients with PF who experience AE are a particularly vulnerable group. Targeted measures to prevent AE could therefore also include physical training or rehabilitations programs aimed at improving body composition, especially PhA. However, these programs must also be incorporated into patients’ everyday lives. Strikingly, more than one third of our patients were engaged in physical activity only once a week or less. Physiotherapy, respiratory therapy, and muscle exercise training were rarely used regularly.

This study provides valuable insights into the frequently unhealthy body composition of patients with PF and possible influencing factors. Notably, it includes different subtypes of PF and collects a wide range of physiological, radiological, and QoL data. Future research should focus on confirming our results in larger cohorts. Integrating BIA measurement into the clinical routine at ILD centres could also help to improve body composition assessment. This could lead to targeted interventions to improve body composition in ILD patients, such as nutrition or rehabilitation programmes. Longitudinal BIA examinations could also help to identify other influencing factors, such as ILD therapies.

However, this study has a number of limitations. Firstly, due to the exploratory design of the study, it is not possible to draw any causal inferences. All findings must be confirmed in greater cohorts. Furthermore, we only included a selected group of PF patients from an ILD expert center. We also included incident and prevalent patients, so that the varying duration of the disease could influence the results, although no statistical correlation could be demonstrated for time since diagnosis. Unfortunately, comparative values for patients aged 70 and over are not available, so we used a comparative cohort of healthy individuals aged between 60 and 69. The subgroups, especially men and women, were not matched, despite similar functional parameters being observed. The underlying rheumatic disease in the SARD-ILD group was not characterized in detail (e.g. joint and organ involvement, disease activity status, etc.), so influences from this cannot be ruled out. Finally, the statistical power of our analyses is clearly limited by the fact that several subgroup analyses are based on relatively small numbers.

## Conclusion

Patients with PF had an unfavourable body composition, characterised by low PhA and cell percentage values, as well as an elevated ECM/BCM index. A higher PhA, ECM/BCM index, and cell percentage was observed at higher FVC values. Secondary analyses revealed significantly lower PhA values in patients with one or more AE and in women. No relevant correlations were found between DLCO, walking distance, FIBI, QoL and BIA parameters. All findings are of explorative and hypothesis-generating nature. A longitudinal and interventional confirmation of our results is needed to guide potential future research directions aimed at improving body composition and outcomes for patients with PF.

## Data Availability

The datasets used and/or analysed during the current study are available from the corresponding author on reasonable request.

## Abbreviations

AE: Acute exacerbation
BCM: Body cell mass
BF: Body fat
BIA: Bioelectrical impedance analysis
COPD: Chronic obstructive pulmonary disease
CT: Computed tomography
DLCO-SB: Diffusion capacity for carbon monoxide single breath
DM: Diabetes mellitus
ECM: Extracellular mass
ECW: Extracellular water
FEV_1_: Forced expiratory volume in one second
FFM: Fat-free mass
FFMI: Fat-free mass index
fHP: Fibrosing hypersensitivity pneumonitis
FIBI: Fibrosis index
FVC: Forced vital capacity
HRCT: High-resolution computed tomography
ICW: Intracellular water
ILD: Interstitial lung diseases
iNSIP: Idiopathic non-specific interstitial pneumonia
IPF: Idiopathic pulmonary fibrosis
IQR: Interquartile range
Kcal: Kilocalories
LBM: Lean body mass
MDT: Multidisciplinary team
mMRC: Modified Medical Research Council Dyspnea Scale
PF: Pulmonary fibrosis
PhA: Phase angle
PPF: Progressive pulmonary fibrosis
QoL: Quality of life
SARD: Systemic autoimmune rheumatic diseases
SD: Standard deviation
SGRQ: St. Georges Respiratory Questionnaire
TBW: Total body water
TLC: Total lung capacity
uILD: Unclassified ILD
YACTA: Yet another CT analyzer
6-MWD: 6-minute walking distance
